# Pre-sleep protein supplementation does not improve performance, body composition, and recovery in British Army recruits (part 1)

**DOI:** 10.3389/fnut.2023.1262044

**Published:** 2023-11-30

**Authors:** Shaun Chapman, Justin Roberts, Andrew J. Roberts, Henry Ogden, Rachel Izard, Lee Smith, Havovi Chichger, Lauren Struszczak, Alex J. Rawcliffe

**Affiliations:** ^1^Army Recruit Health and Performance Research, HQ Army Recruiting and Initial Training Command, Medical Branch, UK Ministry of Defence, Upavon, United Kingdom; ^2^Cambridge Centre for Sport and Exercise Sciences, School of Psychology and Sport Science, Anglia Ruskin University, Cambridge, United Kingdom; ^3^Defence Science and Technology, UK Ministry of Defence, Salisbury, United Kingdom; ^4^Centre for Health, Performance and Wellbeing, Anglia Ruskin University, Cambridge, United Kingdom; ^5^Biomedical Science Research Group, School of Life Science, Anglia Ruskin University, Cambridge, United Kingdom; ^6^Public Health and Sports Sciences, University of Exeter, Exeter, United Kingdom; ^7^Faculty of Science and Engineering, Anglia Ruskin University, Cambridge, United Kingdom

**Keywords:** sport nutrition, strength, fat-free mass, military, diet

## Abstract

Dietary protein is crucial for optimising physical training adaptations such as muscular strength and mass, which are key aims for athletic populations, including British Army recruits. New recruits fail to meet the recommended protein intake during basic training (BT), with negligible amounts consumed in the evening. This study assessed the influence of a daily bolus of protein prior to sleep on performance adaptations, body composition and recovery in British Army recruits. 99 men and 23 women [mean ± standard deviation (SD): age: 21.3 ± 3.5 years, height: 174.8 ± 8.4 cm, body mass 75.4 ± 12.2 kg] were randomised into a dietary control (CON), carbohydrate placebo (PLA), moderate (20 g) protein (MOD) or high (60 g) protein (HIGH) supplementation group. Supplements were isocaloric and were consumed on weekday evenings between 2000 and 2100 for 12 weeks during BT. Performance tests (mid-thigh pull, medicine ball throw, 2 km run time, maximal push-up, and maximal vertical jump) and body composition were assessed at the start and end of BT. Dietary intake, energy expenditure, salivary hormones, urinary nitrogen balance, perceived muscle soreness, rating of perceived exertion, mood, and fatigue were assessed at the start, middle and end of BT. Protein supplementation increased protein intake in HIGH (2.16 ± 0.50 g⸱kg^−1^⸱day^−1^) and MOD (1.71 ± 0.48 g⸱kg^−1^⸱day^−1^) compared to CON (1.17 ± 0.24 g⸱kg^−1^⸱day^−1^) and PLA (1.31 ± 0.29 g⸱kg^−1^⸱day^−1^; *p* < 0.001). Despite this, there was no impact of supplementation on mid-thigh pull performance (CON = 7 ± 19%, PLA = 7 ± 19%, MOD = 0 ± 16%, and HIGH = 4 ± 14%; *p* = 0.554) or any other performance measures (*p* > 0.05). Fat-free mass changes were also similar between groups (CON = 4 ± 3%, PLA = 4 ± 4%, MOD = 3 ± 3%, HIGH = 5 ± 4%, *p* = 0.959). There was no impact of protein supplementation on any other body composition or recovery measure. We conclude no benefits of pre-bed protein supplementation to improve performance, body composition and recovery during BT. It is possible the training stimulus was great enough, limiting the impact of protein supplementation. However, the high degree of inter-participant variability suggests an individualised use of protein supplementation should be explored, particularly in those who consume sub-optimal (<1.6 g⸱kg^−1^⸱day^−1^) habitual amounts of protein.

**Clinical trial registration:** The study was registered with ClinicalTrials.gov, U.S. national institutes (identifier: NCT05998590).

## 1 Introduction

British Army Basic Training (BT) is a physically demanding 14-week course that aims to train civilians into soldiers. The programme includes a variety of arduous activities, such as physical training, strength and conditioning, field exercise, and military-specific tasks (1, 2). BT induces high physiological strain on recruits with daily energy expenditures of ~4,100 and ~ 3,100 kcal·day^−1^ in men and women, respectively (1). It has also been shown that recruits are exposed to high daily external training loads between ~13.5 and ~ 11.8 km·day^−1^ (1). New recruits are required to pass physical performance tests which include a maximal 2 km run, mid-thigh pull and maximal medicine ball throw. As such, nutritional interventions that aim to improve body composition [including fat-free mass (FFM)] and maximise strength can be hypothesised to be advantageous to improve performance and reduce injury risk.

The habitual protein intake of recruits has been observed to be below the current recommendations for military training (1.5–2.0 g·kg^−1^·day^−1^) (3). It has also been established that urinary nitrogen balance (which estimates whole-body protein balance) tends to decline throughout BT (*p* = 0.07) suggesting negative protein balance and sub-optimal protein intakes (4). This, in part, could infer that higher protein intakes result in sustained or positive nitrogen balance, which is pertinent to support skeletal muscle adaptation (5–8). Strategically increasing total daily protein intake during strength training, with the intention to improve maximal strength and FFM has been shown to be an effective strategy (9–11). Furthermore, recent work has shown that protein supplementation prior to sleep, enhances muscle strength and FFM during a 12-week strength training programme in young healthy adults (12). Protein supplementation in the evening time could be particularly beneficial to military recruits due to negligible protein intakes at this time of day (13). It is acknowledged that military training consists of both strength and endurance training. A recent meta-analysis found that general protein supplementation was an effective strategy to support improvements in muscular strength and FFM during arduous concurrent training, including military recruit training (14). More recently, it was found that an additional 40 g of protein as a single bolus per day resulted in a significantly greater decrease in fat-mass compared to an energy matched placebo (protein = 2.1 ± 2.9 kg; placebo = 0.9 ± 2.5 kg) in United States Army recruits (15). As such, protein supplementation in the evening may be a beneficial strategy for military recruits to increase total daily protein intakes and support strength adaptations.

New recruits are required to undertake multiple bouts of exercise each day whilst being exposed to high internal and external training loads (1). There is limited research into the impact of nutritional supplementation on daily physical recovery during military training. In one study in British soldiers, a daily mixed-macronutrient supplement attenuated the reduction in circulating testosterone and cortisol during 8 weeks of arduous training (16). This observation also corresponded to improved physical performance at the end of training compared to a control condition. Conversely, there was no impact of a 40 g protein supplement on hormonal markers of recovery (testosterone and cortisol) compared to a carbohydrate (CHO) placebo in United States Army recruits (15). However, the authors acknowledge that the lack of significant differences between groups may have been due to the high habitual protein intakes in the placebo group (2.2 g⸱kg^−1^⸱day^−1^). Nevertheless, due to previous studies not employing a dietary control (15) and the effects of protein not being isolated against an isocaloric condition (16) the impact of protein supplementation on recovery during military training remains unclear.

The majority of studies investigating the impact of protein supplementation during military training have been conducted in the United States (15, 17–20). These results may not be entirely applicable to British Army recruits due to potential differences in habitual dietary intake, energy expenditure during BT, training entry standards and weekly training loads. Previous investigations have also highlighted a requirement for future research to explore the influence of different forms of macronutrient supplementation (carbohydrate, protein) at various doses in comparison to non-supplemented controls (15). A 20 − 40 g protein dose optimises the stimulation of muscle protein synthesis (MPS) (10) but it is not known if higher doses are beneficial to British Army recruits’ performance outcomes and recovery. Most studies to date have employed a placebo or control group to compare the effects of protein supplementation, meaning the impact of an increased protein intake, independent of energy intake is not known (15, 17–20). This study will provide evidence as to the potential benefit of additional protein intake on the performance outcomes, body composition changes and recovery of British Army recruits which could have important implications on the health and performance of new recruits during BT. Furthermore, this study will also provide evidence on the protein dose needed to optimally support performance, body composition and recovery. To date, there is limited data on protein supplementation during British Army BT and therefore the effects of an additional protein intake in this population is not known. Ultimately, this research will provide evidence on the use of protein supplementation to improve health and performance of new recruits, therefore contributing towards greater military capability and operational readiness of the British Army. The aim of this study was therefore to establish the influence of an isocaloric moderate (20 g) and high (60 g) bolus of protein prior to sleep, on performance adaptations, body composition and chronic recovery in British Army recruits undertaking BT. It was hypothesised that protein supplementation would improve muscular strength [i.e., mid-thigh pull (primary variable)] and FFM (secondary variable) in a dose–response manner.

## 2 Materials and methods

### 2.1 Study design, ethical approval and participants

This randomised controlled trial assigned participants into one of the four dietary supplementation interventions: no nutritional supplement control (CON), carbohydrate placebo (PLA), moderate protein [20 g additional per day (MOD)] or high protein [60 g additional per day (HIGH)]. Data were collected at the start (weeks 1–2), middle (week 6) and end (week 12) of British Army BT. Ethical approval was granted by the United Kingdom Ministry of Defence Research Ethics Committee (1076/MODREC/20) and was conducted in accordance with the Declaration of Helsinki.

In total, 99 men and 23 women [mean ± standard deviation (SD): age: 21.3 ± 3.5 years, height: 174.8 ± 8.4 cm, body mass 75.4 ± 12.2 kg] from four separate intakes between May 2021 and May 2022 completed the study. Participants were randomly allocated into a study condition (CON: *n* = 26; PLA: *n* = 30; MOD: *n* = 32; and HIGH: *n* = 34) using a random number generator. Participants were verbally briefed and provided written-informed consent in week 1 of training at the Army training Centre (Pirbright, Surrey, United Kingdom). Participants were included if they were not taking any other nutritional supplement and were not lactose or dairy intolerant. All participants were passed medically fit to train. A copy of the training programme is not available but previous work in this population has provided a general overview of training activities (1, 4).

*A priori* power analysis was calculated using G*power (Dusseldorf, V 3.1) for a within-and-between factor difference in maximal lower body strength based previously collected data in soldiers receiving a mixed-macronutrient supplement (16). The pre-selected α was 0.05 and β was 0.95. From this calculation, it was determined that a total of 88 participants were required to replicate a partial eta squared (η_p_^2^) of 0.05 (medium).

### 2.2 Supplementation

Participants were administered supplementation each weekday evening between 20:00 and 21:00 h in powder form, mixed fresh with ~500 mL water by a member of the research team. The supplements were isocaloric to isolate the effects of the additional protein intake. The HIGH protein group were provided with 80 g of protein powder, containing 60 g of protein in the form of a concentrate and isolate whey protein blend (Max whey, Maximuscle, Nantwich, United Kingdom). The MOD protein group were provided with the same whey protein blend with a 27 g fixed amount of protein powder which provided 20 g of protein (Maximuscle, max whey, Nantwich, United Kingdom), mixed with 55 g of maltodextrin (Myprotein, 100% maltodextrin carbohydrate, Nantwich, United Kingdom). The PLA group were provided with 35 g of maltodextrin (Myprotein, 100% maltodextrin carbohydrate, Nantwich, United Kingdom), which was bulked up with 35 g of corn flour (buywholefoodsonline, Ramsgate, United Kingdom) and 10 g of Nesquik powder (Nestle, Welwyn Garden city, United Kingdom) The PLA supplement was also flavoured with chocolate and strawberry flavour drops (Myprotein, Flavdrops, Nantwich, United Kingdom) The specific energy and macronutrient breakdown of each supplement was PLA: energy = 286 kcal, CHO = 67 g, protein = 2 g, fat = 1 g; MOD: energy = 286 kcal, CHO = 48 g, protein = 20 g, fat = 2 g; HIGH: energy = 296 kcal, CHO = 8 g, protein = 60 g, and fat = 3 g. The amino acid breakdown of each whey protein supplement was HIGH: leucine = 6.3 g; isoleucine = 4.0 g; valine = 3.8 g; glutamic acid = 10 g; MOD: leucine = 2.1 g; isoleucine = 1.3 g; valine = 1.3 g; and glutamic acid = 3.3 g.

### 2.3 Dietary intake

Energy and macronutrient intake was collected using a self-reported food diary assessed over 4 consecutive days (3 weekdays and 1 weekend day) during weeks 1–2, week 6 and week 12. Participants were verbally instructed to estimate portion size using standardised measures (e.g., one cup, two handfuls, and one palm size) (21), alongside storing any snack or ration discards (i.e., wrappers and packets) to help improve the accuracy of this approach (16). Daily food diaries were checked by a member of the research team the following day to assure compliance and review any unclear information. Food diary records were entered into nutritional analysis software (version 5, Nutritics, Dublin, Ireland) to generate mean daily energy and nutrient intakes using the United Kingdom Scientific Advisory Committee on Nutrition database. Recipes that did not exist in the database (i.e., ration pack foods) were manually entered using the recipe or nutritional content information provided by the caterer.

### 2.4 Nitrogen balance

Prior to the start of collection, participants were issued 3 L urine containers and verbally instructed to collect all urine during a specified 24-h period except the first morning void in line with previously reported research (6, 22–26). Urine was collected over 3 separate non-consecutive days during weeks 1–2, week 6 and week 12 of BT. Urine samples were stored at ambient temperature during the 24-h collection period. Once total urine volume had been recorded, 2 mL aliquots were frozen at −80°C until subsequent analysis.

Urinary urea nitrogen excretion was assessed to determine nitrogen balance using the following equation (6, 27) with protein intake (g) assumed to be 16% nitrogen and miscellaneous nitrogen excretion assumed to be 4 g (28):

$$\begin{array}{l} \mathrm{Nitrogen}\;\\ \mathrm{balance}\;\left(g\right)=\mathrm{total}\;\mathrm{nitrogen}\;\mathrm{intake}\;\left(g\right):\mathrm{urinary}\;\mathrm{urea}\;\mathrm{nitrogen}\;\left(g\right)+4 \end{array}$$

All urinary urea analysis was conducted at the Core Biochemical Assay Laboratory, Addenbrookes Hospital, Cambridge, United Kingdom. Urinary nitrogen was determined enzymatically with a clinical chemistry system (Dimension Flex, Siemens Healthcare Diagnostics Ltd., Camberley, United Kingdom). The method employs a urase/glutamate dehydrogenase coupled technique. Urease specifically hydrolyses urea to form ammonia and carbon dioxide. The ammonia is used by the enzyme glutamate dehydrogenase to reductively aminate α-ketoglutarate with simultaneous oxidation of reduced nicotinamide-adenine dinucleotide. The change in absorption due to the disappearance of nicotinamide adenine dinucleotide (NADH) is directly proportional to the Blood Urea Nitrogen (BUN) concentration in the sample. The intra-and-inter-assay coefficient of variations were 1.2 and 8.8%, respectively.

### 2.5 Energy expenditure

Participants were provided with wrist-based Actigraph watches (WGT3X-BT, Actigraph, Pensacola, United States) on the evening prior to start of each data collection period. In accordance with previous military activity monitoring research, participants were asked to wear the watch on their non-dominant wrist for 24-h and on the same 4 days energy intake was assessed (29). Each participant’s age, stature, and mass (Seca, Hamburg, Germany) were entered into the ActiLife software (v6.13.4, Actigraph, Florida, United States). The Actigraph estimated daily physical activity and duration of time spent in moderate [2,090–6,166 counts per min (cpm)], vigorous (6,167–9,642 cpm), and very vigorous (>9,642 cpm) activity at a 30 Hz sampling rate (30). These activity categories were then be assigned a caloric conversion using the Cooper institutes MET (moderate = 3 METs, vigorous = 6 METs, and very vigorous = 9 METs) to estimate activity energy expenditure (31):

$$\begin{array}{l} \mathrm{Activity}\;\mathrm{energy}\;\mathrm{expenditure}\;\left(\mathrm{kcal}\right)=\mathrm{MET}\;\mathrm{value}\times \mathrm{body}\;\mathrm{mass}\;\left(\mathrm{kg}\right)\times \\ \mathrm{time}\;\left(\min\right) \end{array}$$

Resting energy expenditure (REE) was estimated using the standardised modified Harris-Benedict equation (32) as used previously for military recruits (29):

$$\begin{array}{l} REE\;\left(\mathrm{kcal}\right)=88.362+\left(13.397\times \mathrm{body}\;\mathrm{mass}\;\left(\mathrm{kg}\right)\right)+\\ \left(\begin{array}{l} 4.799\times \\ \mathrm{height}\;\left(\mathrm{cm}\right) \end{array}\right):\left(5.677\times \mathrm{age}\;\left(\mathrm{years}\right)\right) \end{array}$$

Overall daily expenditure was the sum of activity energy expenditure (AEE) and REE. This method has been used to assess the daily energy expenditure of Army recruits undergoing BT in the United States (29).

### 2.6 Recovery

#### 2.6.1 Daily and weekly questionnaires

Daily mood was assessed using the validated Brunel Mood Scale (BRUMS) questionnaire (33) on an online survey platform. The participants were verbally briefed on how to complete the questionnaire and asked to rate a total of 32 emotions from 0 to 4 (zero = not at all, one = a little, two = moderately, three = quite a bit, and four = extremely) at the end of each training day (22:00 h) using an online training diary. The individual emotion scores for the BRUMS questionnaire were then calculated to give a mean score for tension, anger, depression, vigour, fatigue, and confusion in each week. The total global mood score in each week was calculated by summing the scores for tension, depression, anger, fatigue and confusion subtracted from vigour +100 (34).

Subjective measures of the daily rating of perceived exertion (RPE) and global muscle soreness were all recorded at the end of each training day (22:00 h) in weeks 1–2, 6, and 12. These subjective measures were used to indicate daily recovery of the participants and have been used previously in athletic (35) and military cohorts (1). Daily RPE has been shown to be a valid measure of daily internal training load when compared to training impulse data in British military recruits (*R*^2^ = 0.57–0.77) (1). Each subjective measure was recorded as part of the same online training diary as the mood questionnaire and comprised of the 6–20 Borg Scale (RPE) and 0–10 Likert visual scale (global muscle soreness), respectively. At the end of weeks 1–2, 6, and 12 participants completed the Multidimensional Fatigue Syndrome Inventory-Short Form (MFSI-SF) questionnaire. The MFSI-SF is a validated questionnaire which has been used to detect changes in fatigue (36) and has previously been used in Army recruits (37). The MFSI-SF is scored according to a 5-point Likert scale and assesses general fatigue, emotional fatigue, physical fatigue, mental fatigue, and vigour.

### 2.7 Salivary hormones

Saliva was collected from participants within 1 h upon awakening in the morning (06:00 h) prior to breakfast and cleaning teeth. Participants placed an Oral Fluid Collector (OFC) swab (Soma Bioscience, Wallingford, United Kingdom) in their mouth on top of the tongue for ~2 min whilst refraining from talking. The swab was then inserted into a swab storage buffer and stored at 4°C. The samples were then transported to Soma Bioscience, Wallingford for analysis using an Enzyme-Linked Immunosorbent Assay (ELISA) for total testosterone and cortisol. For cortisol and testosterone, a 96-well microlitre plate was coated with Rabbit anti-cortisol antibody (cortisol) or goat anti-mouse IgG (testosterone) at 5 μg·mL^−1^ in phosphate buffered saline and incubated for 90 min at 37°C. The plate was then washed three times with the assay buffer (PBS + 5 mg/mL bovine serum albumin) and blocked with an assay buffer, with 300 μL added per well for 30 min. Duplicate samples (intra-sample CV < 10%) were then added at volumes of 100 μL per well. Horse Radish Peroxide was added (100 μL per well) at a 1:15,000 (cortisol) or 1:18,000 (testosterone) dilution and mixed for 15 min. For testosterone, a monoclonal anti-testosterone antibody was added at 50 ng·mL^−1^ into the assay buffer at 50 μL per well and incubated for 90 min at room temperature (37°C) on a plate shaker. The plates were then washed three times with the assay buffer and Tetramethylbenzidine then added (100 μL) to each well. Following incubation in the dark for 40 min, stop colour development was completed by adding sulphuric acid (100 μL per well) and the resultant optical density read at 450 nm. The intra and inter assay CVs were 7.9 and 9.4% for testosterone and 7.9 and 13.1% for cortisol, respectively.

### 2.8 Body composition

A whole-body scan was performed for the measurement of body composition using Dual X-ray Absorptiometry (DXA; Lunar iDXA, GE Healthcare, United Kingdom) in weeks 1 and 12. Each scan was performed with participants rested and at a similar time of day. Participants were scanned wearing t-shirt and shorts, after removing footwear and jewellery. A whole-body scan provided a three-component model of body composition: fat mass, fat-free soft tissue, and bone mineral content. From these, bone mineral density, fat free mass and percentage body fat were calculated for the determination of body composition on a whole-body level with specific body regions also calculated. For fat-free mass and fat-mass these were arms, legs, truck, android and gynoid. For bone mineral density (BMD) and content these were head, arms, legs, trunk, ribs, pelvis, and spine. Participants were instructed to lie in the supine position on the scanner, face-up and to refrain from moving and talking until the scan was complete. The DXA scanner was calibrated following manufacturer instructions each day prior to use.

### 2.9 Performance

Participants undertook the standardised Army performance tests in weeks 1 and 12 under the supervision of Army physical training instructors. All performance tests were completed with participants wearing Army standardised physical training kit (t-shirt, short, and trainers). Participants were highly motivated because their best effort was required for progression of their military careers (38). Prior to completing the tests, the participants completed a standardised warm-up which was 10 min in duration and followed a Raise, Activate, Mobilise and Potentiate (RAMP) protocol. The parameters measured included aerobic endurance, upper-body muscular endurance, upper-body explosive power, lower-body strength and lower-body explosive power.

To assess aerobic endurance, the time to complete a 2 km best effort run was measured to the nearest second. To assess upper body explosive power, participants completed a seated (with torso at 90°) 4 kg medicine ball throw with the distance thrown recorded in metres to the nearest centimetre using a tape measure. To assess upper-body muscular endurance, participants completed a 2-min maximal press-up test under the supervision of military staff and the research team. Lower-body strength was measured using a mid-thigh pull rig (Absolute performance, Cardiff, United Kingdom) with participants asked to stand in-front of the bar and pull the bar upwards for five seconds. The participant’s highest score from two attempts was taken and measured in kilograms. Explosive lower body power was assessed by a maximal countermovement jump using a jump mat (Takei Scientific Instruments, Tokyo, Japan). Participants were asked to jump as high as possible three times with their hands placed on their hips to prevent upper limb assistance with the highest score recorded (16). Each jump was completed with 30 seconds rest in between jumps.

### 2.10 Statistical analysis

Statistical analyses were carried out using the Statistical Package for Social Sciences (v26, IBM, Armock, New York, United States) with significance set at *p* ≤ 0.05. Data were visually assessed for normality and then quantified using a Shapiro–wilk test. Mixed-model analysis of covariance (ANCOVA) was employed to examine changes in performance outcomes and measures of body composition at week 12 with week 1 data used as the covariate to improve the sensitivity of detecting between group effects, which was the primary research interest (18). A series of ANCOVAs were also used to determine changes in dietary intake, energy expenditure, nitrogen balance and recovery (salivary cortisol and testosterone, subjective measures, and mood). In all instances the week 1 measures were used as covariates. All *post hoc* analyses were undertaken using an adjusted Bonferroni *post hoc* test. Partial eta squared effect sizes were classified as small (ƞ^2^ = 0.01), medium (ƞ^2^ = 0.0.06) and large (ƞ^2^ = 0.14) (16, 39). Data are presented as mean ± SD.

## 3 Results

### 3.1 Dietary intake

Nutritional supplementation (in any form) increased absolute energy intake in PLA, MOD and HIGH compared to CON during week 12 of BT (*F*[3,93] = 3.940, *p* = 0.011, ƞ_p_^2^ = 0.113) but not during week 6 (*F*[1,93] = 18.260, *p* = 0.132, ƞ_p_^2^ = 0.058; Table 1). Specifically, *post hoc* analysis revealed nutritional supplementation increased energy intake by *circa* 500 kcal in PLA (*p* = 0.038), MOD (*p* = 0.022) and HIGH (*p* = 0.015), in comparison to CON. When calculated relative to body mass, supplementation increased energy intake in the supplement groups week 12 (*F*[3,91] = 3.078, *p* = 0.031, ƞ_p_^2^ = 0.092) but not week 6 (*F*[3,91] = 1.579, *p* = 0.200, ƞ_p_^2^ = 0.049; Table 2). Energy intake was greater but did not reach significance in PLA (*p* = 0.056), MOD (*p* = 0.055) and HIGH (*p* = 0.060) compared to CON at week 12 (Table 2).

**Table 1:** Absolute daily dietary intake in each group in weeks 1, 6, and 12.

Nutrient	Group	Week 1	Week 6	Week 12
Energy (Kcal)	CON	2335 ± 517	2281 ± 398	2146. ± 393^b,c,d^
	PLA	2209 ± 503	2625 ± 509	2530 ± 617^a^
	MOD	2373 ± 456	2561 ± 527	2632 ± 591^a^
	HIGH	2392 ± 511	2482 ± 629	2648 ± 475^a^
CHO (g)	CON	269 ± 68	268 ± 69^b^	246 ± 49^b^
	PLA	236 ± 48	337 ± 65^a,d^	331 ± 84^a,d^
	MOD	252 ± 59	313 ± 74	322 ± 82
	HIGH	270 ± 67	266 ± 93^b^	278 ± 74^b^
Protein (g)	CON	113 ± 19	100 ± 16^c,d^	91 ± 18^c,d^
	PLA	114 ± 32	102 ± 22^c,d^	98 ± 21^c,d^
	MOD	118 ± 31	125 ± 29^a,b,d^	125 ± 26^a,b,d^
	HIGH	114 ± 20	154 ± 25^a,b,c^	156 ± 17^a,b,c^
Fat (g)	CON	89 ± 27	89 ± 16	88 ± 22
	PLA	89 ± 27	96 ± 26	90 ± 28
	MOD	98 ± 26	89 ± 21	94 ± 27
	HIGH	94 ± 26	88 ± 26	100 ± 22

Data shown as mean ± standard deviation. g, grams; kg, kilograms; d, days; CHO, Carbohydrate; ^a^Different vs. CON; ^b^Different vs. PLA; ^c^Different vs. MOD; and ^d^Different vs. HIGH.

**Table 2:** Dietary intake relative to body mass (kg) in each group in weeks 1, 6 and 12.

Nutrient	Group	Week 1	Week 6	Week 12
Energy (kcal⸱kg^−1^⸱day^−1^)	CON	29.63 ± 7.95	29.24 ± 6.62	27.59 ± 6.28
	PLA	29.38 ± 6.22	35.25 ± 7.47	34.00 ± 9.11
	MOD	32.36 ± 7.79	34.93 ± 8.97	36.15 ± 11.31
	HIGH	32.70 ± 8.56	34.47 ± 13.94	36.17 ± 8.79
CHO (g⸱kg^−1^⸱day^−1^)	CON	3.38 ± 0.97	3.41 ± 1.07^b^	3.18 ± 0.86^b^
	PLA	3.14 ± 0.60	4.54 ± 1.02^a,d^	4.48 ± 1.31^a,c^
	MOD	3.47 ± 1.09	4.28 ± 1.24	4.44 ± 1.57^b,d^
	HIGH	3.68 ± 1.02	3.74 ± 1.97^b^	3.80 ± 1.15^c^
Protein (g⸱kg^−1^⸱day^−1^)	CON	1.44 ± 0.32	1.27 ± 0.28^c,d^	1.17 ± 0.24^c,d^
	PLA	1.53 ± 0.45	1.37 ± 0.30^c,d^	1.31 ± 0.29^c,d^
	MOD	1.59 ± 0.41	1.70 ± 0.40^b,c d^	1.71 ± 0.48^a,b,d^
	HIGH	1.58 ± 0.37	2.13 ± 0.65^a,b,c^	2.16 ± 0.50^a,b,c^
Fat (g⸱kg^−1^⸱day^−1^)	CON	1.15 ± 0.39	1.16 ± 0.26	1.13 ± 0.31
	PLA	1.18 ± 0.33	1.29 ± 0.35	1.20 ± 0.38
	MOD	1.34 ± 0.41	1.22 ± 0.38	1.29 ± 0.48
	HIGH	1.30 ± 0.42	1.22 ± 0.48	1.38 ± 0.37

Data shown as mean ± standard deviation. g, grams; kg, kilograms; d, days; CHO, Carbohydrate; ^a^Difference vs. CON; ^b^Difference vs. PLA; ^c^Difference vs. MOD; and ^d^Difference vs. HIGH.

For CHO intake, there was a significant group interaction at week 6 (*F*[3,93] = 5.795, *p* = 0.001, ƞ_p_^2^ = 0.157) and 12 (*F*[3,93] = 8.263, *p* < 0.001, ƞ_p_^2^ = 0.210; Table 1). Supplementation resulted in greater CHO intake in PLA compared to CON (*p* = 0.017) and HIGH (*p* = 0.002) at week 6 and in PLA compared to CON (*p* < 0.001) and HIGH (*p* = 0.006) at week 12. When intakes were calculated relative to body mass, there was also a significant group interaction at week 6 (*F*[3,91] = 4.439, *p* = 0.006, ƞ_p_^2^ = 0.128) and 12 (*F*[3,91] = 8.278, *p* < 0.001, ƞ_p_^2^ = 0.214; Table 2). CHO intake was greater at week 6 in PLA compared to CON (*p* = 0.024) and HIGH (*p* = 0.018). CHO intake was greater at week 12 in PLA compared to CON (*p* < 0.001) and in MOD compared to CON (*p* = 0.006) and HIGH (*p* = 0.047).

Supplementation resulted in a significant group interaction for self-reported protein intake at weeks 6 (*F*[3,93] = 31.416, *p* < 0.001, ƞ_p_^2^ = 0.503) and 12 (*F*[1,93] = 55.910, *p* < 0.001, ƞ_p_^2^ = 0.643; Table 1). Specifically, protein supplementation resulted in greater protein intake in HIGH compared to CON (*p* < 0.001), PLA (*p* < 0.001) and MOD (*p* < 0.001). Protein supplementation also resulted in greater protein intake in MOD compared to CON (*p* = 0.010) and PLA (*p* = 0.005) at week 6. By week 12, protein supplementation resulted in greater total daily protein intake in HIGH compared to CON (*p* < 0.001), PLA (*p* < 0.001) and MOD (*p* < 0.001). Protein intake was also greater at week 12 in MOD compared to CON (*p* < 0.001) and PLA (*p* < 0.001; Table 1).

When protein intakes were expressed relative to body mass, there was an interaction between groups at weeks 6 (*F*[3,91] = 20.98, *p* < 0.001, ƞ_p_^2^ = 0.404) and 12 (*F*[3,91] = 31.867, *p* < 0.001, ƞ_p_^2^ = 0.512; Table 2). Supplementation resulted in greater protein intake in HIGH compared to CON (*p* < 0.001), PLA (*p* < 0.001) and MOD (*p* < 0.001). Protein intake was also greater in MOD compared to PLA (*p* = 0.042) and CON (*p* < 0.001). At week 12, protein intake was also greater in HIGH compared to CON (*p* < 0.001), PLA (*p* < 0.001) and MOD (*p* < 0.001) and in MOD compared to CON (*p* < 0.001) and PLA (*p* = 0.002; Table 2).

There were no group interactions for absolute fat intake at week 6 (*F*[3,93] = 1.028, *p* = 0.384, ƞ_p_^2^ = 0.032) or 12 (*F*[1,93] = 1.002, *p* = 0.395, ƞ_p_^2^ = 0.031) indicating relative consistency between groups (Table 1). Similarly, there were no group interactions for fat intake when intakes were expressed relative to body mass at weeks 6 (*F*[3,91] = 0.952, *p* = 0.419, ƞ_p_^2^ = 0.030) or 12 (*F*[3,91] = 0.940, *p* = 0.425, ƞ_p_^2^ = 0.030; Table 2).

### 3.2 Energy expenditure

There was no group interactions for energy expenditure at week 6 (*F*[3,65] = 0.829, *p* = 0.483, ƞ_p_^2^ = 0.037) or week 12 (*F*[3,65] = 0.912, *p* = 0.440, ƞ_p_^2^ = 0.040; Table 3).

**Table 3:** Energy expenditure in each group at weeks 1, 6 and 12.

Measure	Group	Week 1	Week 6	Week 12
Energy expenditure (kcal⸱day^−1^)	CON	3662 ± 549	3584 ± 623	3425 ± 616
	PLA	3837 ± 825	3564 ± 799	3623 ± 667
	MOD	3762 ± 767	3515 ± 958	3667 ± 814
	HIGH	3896 ± 843	3834 ± 651	3669 ± 632

Data presented as mean ± SD. CON, Control; PLA, Placebo; MOD, Moderate protein; and HIGH, High protein.

### 3.3 Nitrogen balance

There was a group interaction for nitrogen excretion at week 6 (*F*[3,40] = 7.875, *p* < 0.001, ƞ_p_^2^ = 0.355) but not week 12 (*F*[1,40] = 3.102, *p* = 0.085, ƞ_p_^2^ = 0.141). *Post hoc* analysis revealed greater nitrogen excretion in HIGH compared to CON (16.9 ± 4.9 vs. 11.4 ± 3.5 g⸱day^−1^, *p* = 0.011), PLA (10.6 ± 2.6 g⸱day^−1^, *p* < 0.001) and MOD (13.2 ± 4.0 g⸱day^−1^, *p* = 0.022) at week 6.

**Figure 1:**
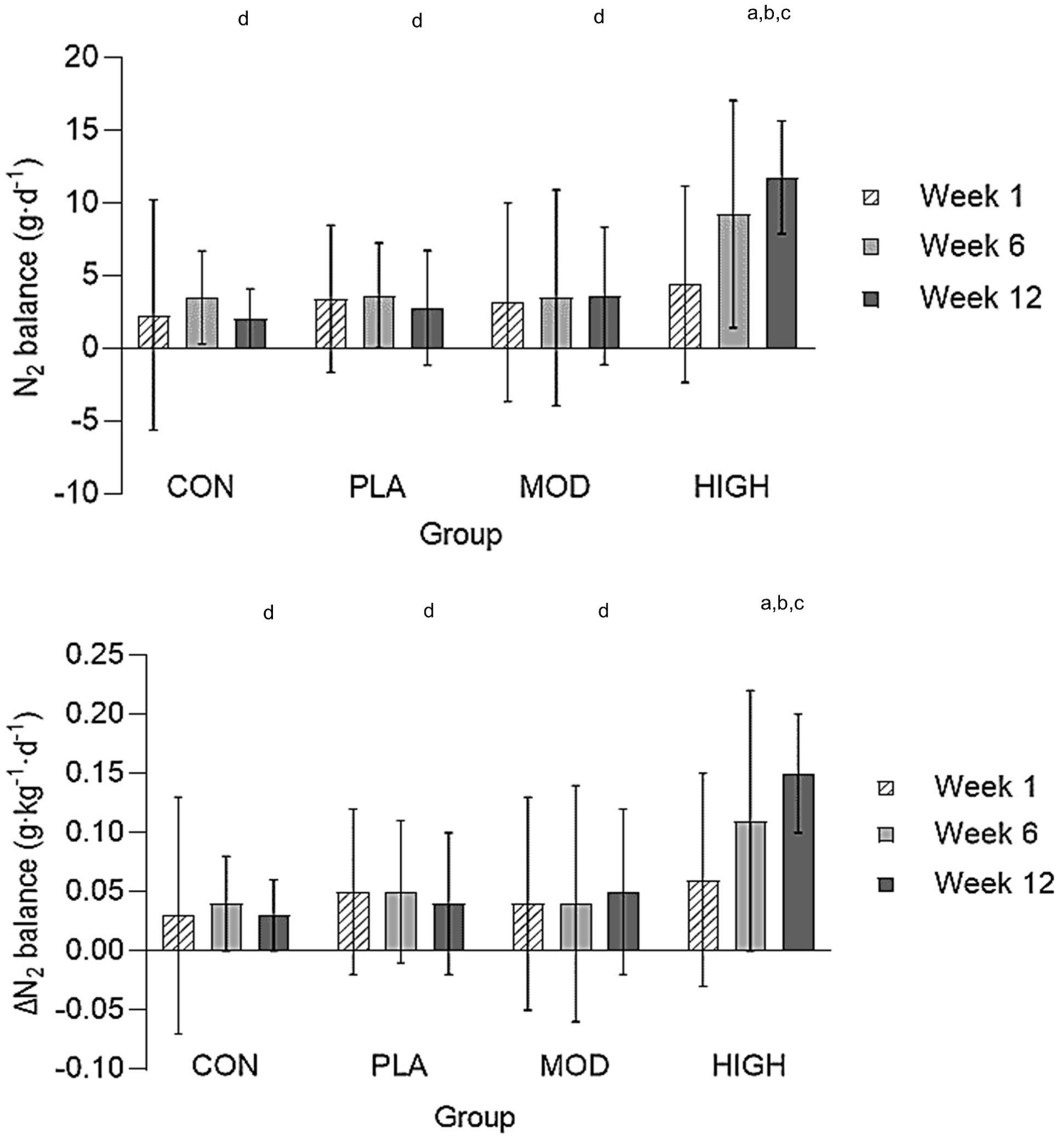
Nitrogen balance in all groups in weeks 1, 6, and 12. Absolute nitrogen balance (top) and relative nitrogen balance (bottom). Data shown as mean ± standard deviation. g, grams; kg, kilograms; N, Nitrogen; CON, Control; PLA, Placebo; MOD, Moderate protein; and HIGH, High protein. ^a^Difference vs. CON; ^b^Difference vs. PLA; ^c^Difference vs. MOD; and ^d^Difference vs. HIGH.

Following supplementation there was a significant group interaction for nitrogen intake at week 6 (*F*[1,43] = 34.434, *p* < 0.001, ƞ_p_^2^ = 0.706) and week 12 (*F*[1,43] = 62.197, *p* < 0.001, ƞ_p_^2^ = 0.813). *Post hoc* analysis revealed that nitrogen intake was greater in HIGH compared to CON (24.9 ± 4.1 vs. 15.0 ± 2.2 g⸱day^−1^, *p* < 0.001), PLA (14.5 ± 2.2 g⸱day^−1^, *p* < 0.001) and MOD (18.7 ± 2.2 g⸱day^−1^, *p* < 0.001) and greater in MOD compared to PLA (*p* = 0.007) at week 6. Nitrogen intake was also greater in HIGH compared to CON (25.2 ± 2.9 vs. 14.1 ± 1.8 g⸱day^−1^, *p* < 0.001), PLA (14.2 ± 1.8 g⸱day^−1^, *p* < 0.001) and MOD (16.6 ± 2.9 g⸱day^−1^, *p* < 0.001) at week 12. The greater nitrogen intake in the protein supplementation groups resulted in a group interaction for nitrogen balance at weeks 6 (*F*[3,43] = 2.200, *p* = 0.102, ƞ_p_^2^ = 0.133) and 12 (*F*[3,43] = 17.650, *p* < 0.001, ƞ_p_^2^ = 0.552; Figure 1). Nitrogen balance was greater in HIGH compared to CON (*p* < 0.001), PLA (*p* < 0.001) and MOD (*p* < 0.001) at week 12 (Figure 1).

**Figure 2:**
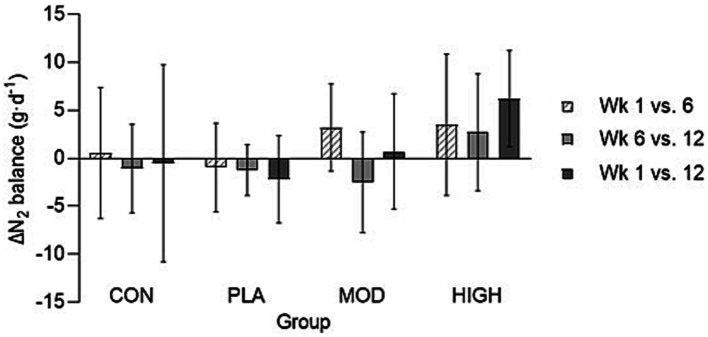
The change in (∆) absolute nitrogen balance in all groups in weeks 1, 6, and 12. Data shown as mean ± standard deviation. g, grams; kg, kilograms; N, Nitrogen; CON, Control; PLA, Placebo; MOD, Moderate protein; and HIGH, High protein. ^a^Difference vs. CON; ^b^Difference vs. PLA; ^c^Difference vs. MOD; and ^d^Difference vs. HIGH.

When expressed relative to body mass, there was no group interaction for nitrogen balance at week 6 (*F*[3,43] = 2.187, *p* = 0.103, ƞ_p_^2^ = 0.103) but there was at week 12 (*F*[3,43] = 15.738, *p* < 0.001, ƞ_p_^2^ = 0.523). Nitrogen balance was greater in HIGH compared to CON (*p* < 0.001), PLA (*p* < 0.001) and MOD (*p* < 0.001) at week 12 (Figure 2).

### 3.4 Performance

**Figure 3:**
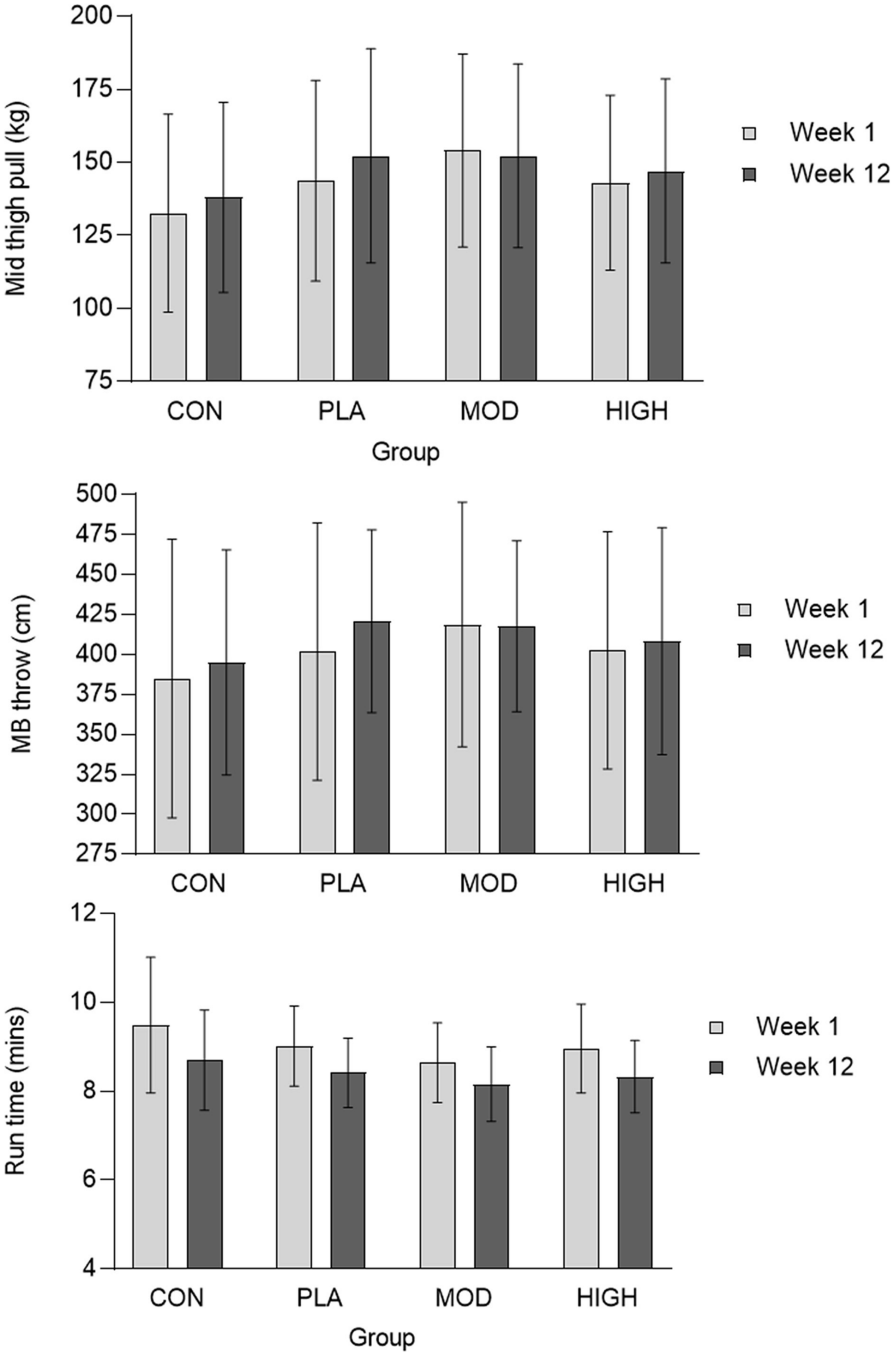
Mid-thigh pull (top), medicine ball throw (middle), and run (bottom) performance data for each group. Data presented as mean ± standard deviation. Kg, kilograms; cm, centimetres; MB, Medicine ball. CON, Control; PLA, Placebo; MOD, Moderate protein; HIGH, High protein; and min, Minutes.

**Figure 4:**
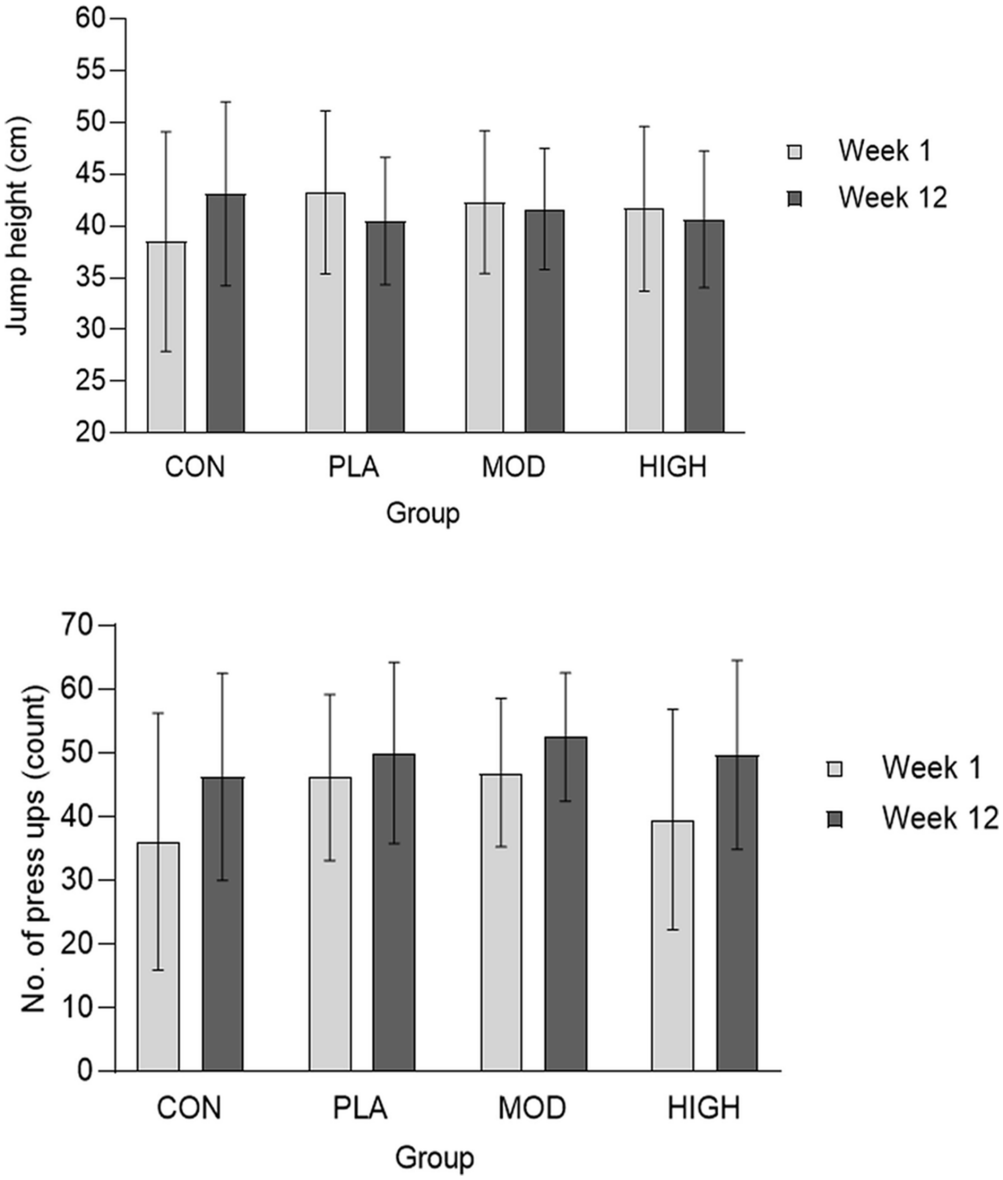
Jump height (top) and press-up (bottom) performance at week 1 and 12 in each group. Data presented as mean ± SD. CON, Control; PLA, Placebo; MOD, Moderate protein; and HIGH, High protein.

There was no effect of supplementation for mid-thigh pull (*F*[3,107] = 0.701, *p* = 0.554, ƞ_p_^2^ = 0.021), medicine ball throw (*F*[3,107] = 1.143, *p* = 0.335, ƞ_p_^2^ = 0.033), run time (*F*[3,95] = 0.214, *p* = 0.886, ƞ_p_^2^ = 0.007), press-up (*F*[3,92] = 1.180, *p* = 0.322, ƞ_p_^2^ = 0.040) or jump performance (*F*[3,90] = 1.165, *p* = 0.328, ƞ_p_^2^ = 0.040; Figures 3, 4; Table 4).

**Table 4:** Performance parameters in each group.

Measure	Group	% Change
Mid-thigh pull (kg)	CON	7 ± 19
	PLA	7 ± 13
	MOD	0 ± 16
	HIGH	4 ± 14
Medicine ball throw (cm)	CON	7 ± 34
	PLA	10 ± 35
	MOD	1 ± 11
	HIGH	3 ± 15
No. of press-ups	CON	5 ± 9
	PLA	4 ± 10
	MOD	4 ± 6
	HIGH	6 ± 12
Jump height (cm)	CON	5 ± 15
	PLA	−4 ± 8
	MOD	−1 ± 9
	HIGH	−2 ± 7
2 km run (secs)	CON	−8 ± 7
	PLA	−6 ± 7
	MOD	−5 ± 6
	HIGH	−7 ± 5

Data presented as mean ± standard deviation. %, Percentage; CON, Control; PLA, Placebo, MOD, Moderate; and HIGH, High.

### 3.5 Body composition

There was no impact of supplementation on body mass (*F*[3,121] = 0.400, *p* = 0.753, ƞ^2^ = 0.010). The descriptive data for each group in week 1 and 12 were as follows: CON = 76.5 ± 11.8 vs. 76.2 ± 9.3 kg; HIGH = 75.4 ± 12.4 vs. 75.6 ± 11.1 kg; MOD = 74.8 ± 12.5 vs. 74.9 ± 10.5 kg; and PLA = 74.6 ± 11.9 vs. 74.7 ± 10.4 kg.

#### 3.5.1 Fat-free-mass

There was no effect of supplementation for total FFM (*F*[3,121] = 0.193, *p* = 0.901, ƞ_p_^2^ = 0.005; Table 5). There was no impact of protein supplementation on android (*F*[3,121] = 1.262, *p* = 0.291, ƞ_p_^2^ = 0.032), arms (*F*[3,121] = 0.607, *p* = 0.612, ƞ_p_^2^ = 0.016), gynoid (*F*[3,121] = 0.965, *p* = 0.412, ƞ_p_^2^ = 0.025), legs (*F*[3,121] = 0.238, *p* = 0.870, ƞ_p_^2^ = 0.006) or trunk (*F*[3,121] = 0.298, *p* = 0.827, ƞ_p_^2^ = 0.008) FFM (Table 5).

**Table 5:** Fat-free mass and fat-mass in week 1 and 12 in each group.

Body region	Group	FFM Week 1	FFM week 12	% Change	FM week 1	FM week 12	% Change
Total (kg)	CON	57.5 ± 9.3	60.0 ± 9.1	4 ± 3	18.9 ± 8.2	16.2 ± 5.7	−9 ± 21
	PLA	58.1 ± 9.3	59.9 ± 8.8	4 ± 4	16.5 ± 6.5	14.8 ± 5.0	−7 ± 20
	MOD	59.4 ± 9.4	61.3 ± 9.0	3 ± 3	15.4 ± 6.0	13.8 ± 4.3	−6 ± 18
	HIGH	58.1 ± 10.3	60.6 ± 9.9	5 ± 4	17.3 ± 6.5	15.0 ± 5.0	−9 ± 23
Android (kg)	CON	3.6 ± 0.7	3.9 ± 0.7	8 ± 5	1.3 ± 0.8	1.0 ± 0.5	−11 ± 41
	PLA	3.7 ± 0.6	3.8 ± 0.5	5 ± 6	1.1 ± 0.7	0.9 ± 0.5	−10 ± 32
	MOD	3.8 ± 0.6	3.9 ± 0.7	5 ± 4	1.0 ± 0.6	0.8 ± 0.4	−9 ± 33
	HIGH	3.7 ± 0.6	3.9 ± 0.3	7 ± 7	1.2 ± 0.7	0.9 ± 0.5	−10 ± 46
Arms (kg)	CON	6.9 ± 1.8	7.2 ± 1.6	5 ± 6	2.2 ± 0.9	1.8 ± 0.6	−11 ± 14
	PLA	7.4 ± 1.8	7.6 ± 1.7	3 ± 9	1.9 ± 0.6	1.7 ± 0.5	−10 ± 14
	MOD	7.4 ± 1.6	7.6 ± 1.5	4 ± 5	1.7 ± 0.7	1.6 ± 0.5	−3 ± 18
	HIGH	7.2 ± 1.8	7.5 ± 1.8	5 ± 6	1.9 ± 0.7	1.7 ± 0.6	−7 ± 20
Gynoid (kg)	CON	8.8 ± 1.4	9.4 ± 1.4	6 ± 5	3.3 ± 1.5	2.9 ± 1.3	−9 ± 22
	PLA	8.9 ± 1.5	9.2 ± 1.4	4 ± 5	2.9 ± 1.2	2.6 ± 10.3	−6 ± 21
	MOD	9.1 ± 1.5	9.5 ± 1.6	4 ± 5	2.7 ± 1.1	2.3 ± 0.8	−8 ± 21
	HIGH	9.0 ± 1.7	9.5 ± 1.6	6 ± 5	3.0 ± 1.2	2.6 ± 0.9	−8 ± 24
Legs (kg)	CON	20.5 ± 3.3	21.1 ± 3.3	3 ± 4	6.9 ± 3.1	6.2 ± 2.6	−6 ± 18
	PLA	20.6 ± 3.7	21.3 ± 3.5	3 ± 5	5.9 ± 2.3	5.5 ± 1.9	−4 ± 20
	MOD	21.0 ± 3.8	21.6 ± 3.5	3 ± 5	5.3 ± 2.0	5.0 ± 1.6	−1 ± 18
	HIGH	20.6 ± 3.9	21.3 ± 3.7	4 ± 5	6.1 ± 2.3	5.6 ± 1.9	−6 ± 18
Trunk (kg)	CON	26.1 ± 4.3	27.7 ± 4.4	6 ± 5	8.9 ± 4.5	7.3 ± 2.8	−12 ± 29
	PLA	26.0 ± 3.9	27.2 ± 3.7	5 ± 4	7.8 ± 3.6	6.7 ± 2.7	−8 ± 26
	MOD	27.1 ± 4.1	28.1 ± 4.1	4 ± 3	7.4 ± 3.7	6.3 ± 2.5	−9 ± 23
	HIGH	26.3 ± 4.5	27.9 ± 4.4	6 ± 4	8.3 ± 3.8	6.8 ± 2.9	−11 ± 32

Data presented as mean ± standard deviation. FFM, Fat-free mass; FM, Fat-mass; % change, Percent change; and kg, kilograms.

#### 3.5.2 Fat-mass

There was no effect of supplementation for total (*F*[3,121] = 0.101, *p* = 0.959, ƞ_p_^2^ = 0.003), android (*F*[3,121] = 0.966, *p* = 0.412, ƞ_p_^2^ = 0.025), arms (*F*[3,121] = 0.236, *p* = 871, ƞ_p_^2^ = 0.006), gynoid (*F*[3,121] = 0.235, *p* = 0.872, ƞ_p_^2^ = 0.006), legs (*F*[3,121] = 0.148, *p* = 0.931, ƞ_p_^2^ = 0.004) or trunk (*F*[3,121] = 0.600, *p* = 0.616, ƞ_p_^2^ = 0.016) fat mass (Table 5).

#### 3.5.3 Bone mineral density

There was no effect of supplementation for total (*F*[3,121] = 0.274, *p* = 0.844, ƞ_p_^2^ = 0.007), head (*F*[3,121] = 0.114, *p* = 0.952, ƞ_p_^2^ = 0.003), arms (*F*[3,121] = 0.255, *p* = 0.858, ƞ_p_^2^ = 0.007), legs (*F*[3,121] = 0.901, *p* = 0.443, ƞ_p_^2^ = 0.023), trunk (*F*[3,121] = 0.775, *p* = 0.510, ƞ_p_^2^ = 0.020), ribs (*F*[3,121] = 0.360, *p* = 0.782, ƞ_p_^2^ = 0.009), pelvis (*F*[3,121] = 0.389, *p* = 0.761, ƞ_p_^2^ = 0.010) or spine (*F*[3,121] = 1.739, *p* = 0.163, ƞ_p_^2^ = 0.044) BMD (Table 6).

**Table 6:** Bone parameters in each group at week 1 and 12.

Region	Group	BMD week 1 (g⸱cm^2^)	BMD week 12 (g⸱cm^2^)	%	BMC week 1 (g)	BMC week 12 (g)	%
Total	CON	1.21 ± 0.10	1.23 ± 0.10	1 ± 2	2,840 ± 406	2,852 ± 395	0 ± 1
	PLA	1.25 ± 0.12	1.28 ± 0.12	3 ± 4	2,887 ± 441	2,923 ± 448	1 ± 4
	MOD	1.30 ± 0.12	1.32 ± 0.11	2 ± 2	3,034 ± 466	3,049 ± 465	1 ± 1
	HIGH	1.27 ± 0.15	1.29 ± 0.13	2 ± 2	2,978 ± 496	2,990 ± 493	0 ± 1
Head	CON	2.12 ± 0.26	2.14 ± 0.25	1 ± 2	515 ± 64	516 ± 65	0 ± 0
	PLA	2.18 ± 0.23	2.20 ± 0.26	1 ± 2	527 ± 65	530 ± 66	1 ± 2
	MOD	2.18 ± 0.23	2.21 ± 0.23	1 ± 3	529 ± 60	531 ± 60	0 ± 1
	HIGH	2.17 ± 0.29	2.20 ± 0.29	1 ± 2	530 ± 67	533 ± 70	0 ± 2
Arms	CON	0.91 ± 0.10	0.93 ± 0.10	2 ± 10	395 ± 80	399 ± 79	1 ± 3
	PLA	0.93 ± 0.10	0.99 ± 0.12	7 ± 14	411 ± 79	414 ± 76	1 ± 7
	MOD	0.97 ± 0.12	1.00 ± 0.12	4 ± 9	425 ± 76	428 ± 72	1 ± 3
	HIGH	0.94 ± 0.14	0.98 ± 0.11	6 ± 10	419 ± 87	420 ± 88	0 ± 3
Legs	CON	1.29 ± 0.10	1.31 ± 0.12	2 ± 2	1,069 ± 165	1,080 ± 157	1 ± 1
	PLA	1.34 ± 0.16	1.37 ± 0.16	2 ± 4	1,096 ± 213	1,114 ± 214	2 ± 4
	MOD	1.40 ± 0.14	1.41 ± 0.14	1 ± 2	1,152 ± 184	1,166 ± 183	1 ± 1
	HIGH	1.37 ± 0.17	1.38 ± 0.17	1 ± 2	1,131 ± 218	1,142 ± 218	1 ± 1
Trunk	CON	1.03 ± 0.11	1.03 ± 0.10	0 ± 2	859 ± 170	856 ± 163	0 ± 5
	PLA	1.05 ± 0.11	1.06 ± 0.11	1 ± 4	852 ± 151	864 ± 157	1 ± 5
	MOD	1.11 ± 0.11	0.12 ± 0.11	0 ± 2	927 ± 184	922 ± 186	−1 ± 3
	HIGH	1.07 ± 0.13	1.08 ± 0.12	0 ± 2	897 ± 176	893 ± 169	0 ± 4
Ribs	CON	0.93 ± 0.10	0.91 ± 0.09	−2 ± 4	292 ± 62	276 ± 59	−5 ± 9
	PLA	0.93 ± 0.10	0.93 ± 0.09	0 ± 4	278 ± 58	277 ± 55	0 ± 8
	MOD	0.96 ± 0.11	0.96 ± 0.11	−1 ± 4	298 ± 75	288 ± 69	−3 ± 5
	HIGH	0.95 ± 0.13	0.93 ± 0.12	−2 ± 4	299 ± 72	285 ± 65	−4 ± 9
Pelvis	CON	1.08 ± 0.13	1.10 ± 0.13	2 ± 2	366 ± 77	381 ± 74	5 ± 6
	PLA	1.11 ± 0.14	1.14 ± 0.14	2 ± 5	376 ± 77	388 ± 78	3 ± 5
	MOD	1.20 ± 0.13	1.22 ± 0.13	1 ± 2	413 ± 76	421 ± 78	2 ± 3
	HIGH	1.15 ± 0.16	1.17 ± 0.15	1 ± 2	392 ± 79	402 ± 79	3 ± 4
Spine	CON	1.10 ± 0.13	1.10 ± 0.12	0 ± 3	201 ± 40	197.94 ± 38	−1 ± 6
	PLA	1.11 ± 0.13	0.13 ± 0.14	1 ± 4	197 ± 34	198.24 ± 37	0 ± 6
	MOD	1.18 ± 0.14	1.19 ± 0.15	1 ± 3	215 ± 46	213.20 ± 52	−1 ± 11
	HIGH	1.13 ± 0.13	1.14 ± 0.12	0 ± 2	205 ± 36	205.70 ± 36	0 ± 6

Data presented as mean ± standard deviation. BMC, Bone mineral content; BMD, Bone mineral density; %, Percent change; cm, Centimetres; g. grams; CON, Control; PLA, Placebo; MOD, Moderate; and HIGH, High.

#### 3.5.4 Bone mineral content

There was no effect of supplementation group for total (*F*[3,121] = 0.177, *p* = 0.911, ƞ_p_^2^ = 0.005), head (*F*[3,121] = 0.928, *p* = 0.430, ƞ_p_^2^ = 0.024), arms (*F*[3,121] = 1.173, *p* = 0.323, ƞ_p_^2^ = 0.030), legs (*F*[3,121] = 0.568, *p* = 0.637, ƞ_p_^2^ = 0.015), trunk (*F*[3,121] = 0.601, *p* = 0.616, ƞ_p_^2^ = 0.016), ribs (*F*[3,121] = 0.138, *p* = 0.937, ƞ_p_^2^ = 0.004), pelvis (*F*[3,121] = 0.832, *p* = 0.479, ƞ_p_^2^ = 0.021) or spine (*F*[3,121] = 0.810, *p* = 0.491, ƞ_p_^2^ = 0.021) BMC (Table 6).

### 3.6 Salivary hormones

**Figure 5:**
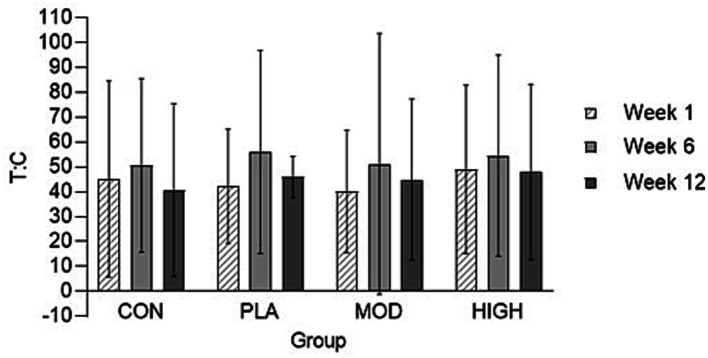
Salivary testosterone: cortisol (T: C) ratio in each group at weeks 1, 6, and 12. Data presented as mean ± standard deviation. CON, Control; HIGH, High protein; MOD, Moderate protein; and PLA, Placebo.

**Figure 6:**
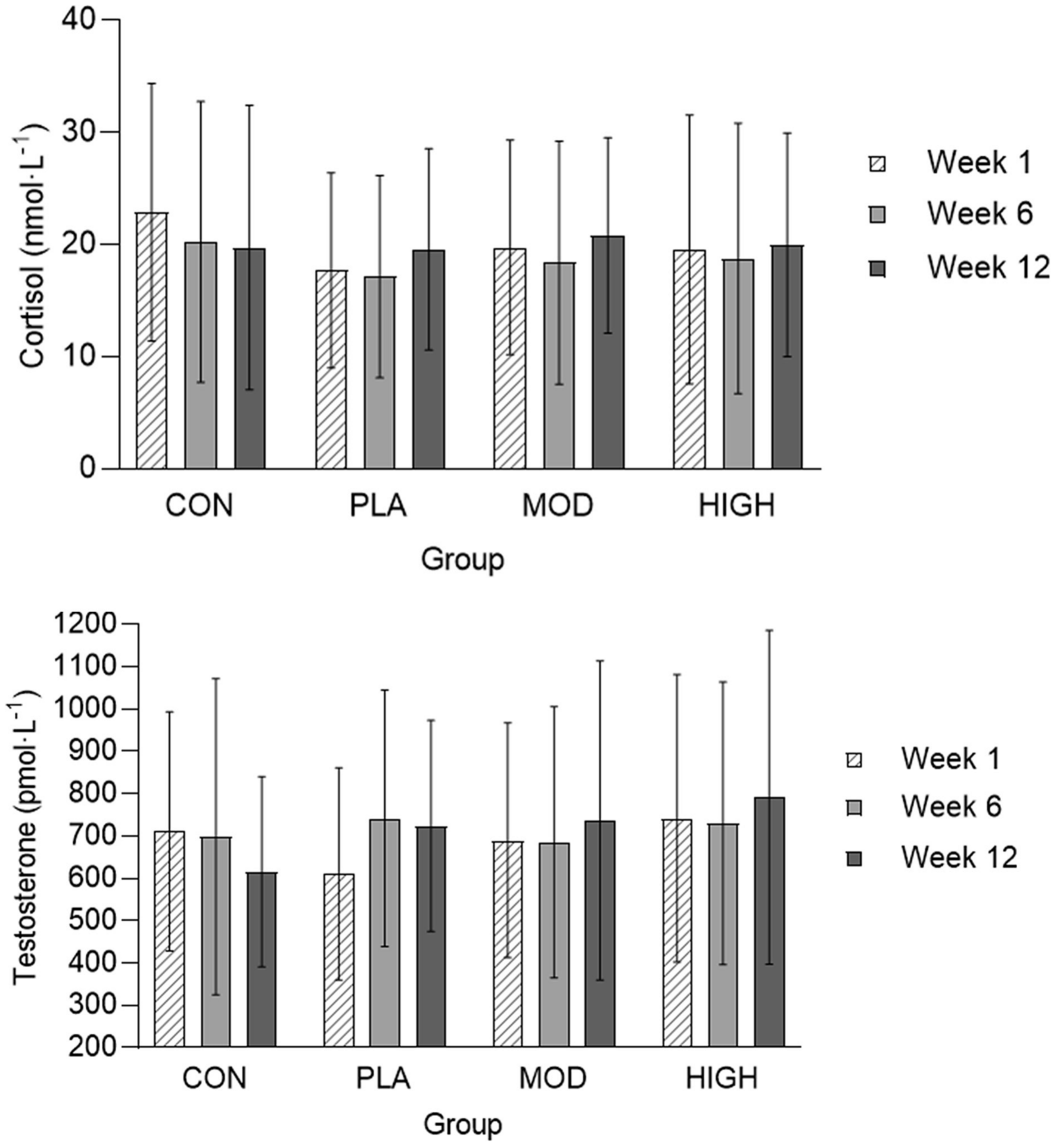
Salivary cortisol and testosterone in each group at weeks 1, 6, and 12. Data presented as mean ± standard deviation. CON, Control; HIGH, High protein; MOD, Moderate protein; and PLA, Placebo.

The salivary cortisol and testosterone concentrations for each group are shown in Figures 5, 6. There was no effect of supplementation on cortisol at week 6 (*F*[3,116] = 0.021, *p* = 0.996, ƞ_p_^2^ = 0.001) or 12 (*F*[3,116] = 1.514, *p* = 0.674, ƞ_p_^2^ = 0.013). There was also no effect of supplementation on testosterone at week 6 [*F*(3,113] = 0.812, *p* = 0.490, ƞ_p_^2^ = 0.021) and 12 (*F*[3,113] = 2.329, *p* = 0.078, ƞ_p_^2^ = 0.058) or on the T:C ratio in week 6 (*F*[3,110] = 0.261, *p* = 0.853, ƞ_p_^2^ = 0.007) or week 12 (*F*[3,110] = 0.438, *p* = 0.726, ƞ_p_^2^ = 0.012).

### 3.7 Daily muscle soreness and RPE

There was no effect of supplementation for muscle soreness at week 6 (*F*[3,43] = 0.520, *p* = 0.670, ƞ_p_^2^ = 0.035) and week 12 (*F*[3,43] = 0.313, *p* = 0.816, ƞ_p_^2^ = 0.021) or RPE at week 6 (*F*[3,36] = 2.450, *p* = 0.079, ƞ_p_^2^ = 0.170) and week 12 (*F*[3,36] = 1.602, *p* = 0.091, ƞ_p_^2^ = 0.065; Table 7).

**Table 7:** Daily RPE and muscle soreness in each group at weeks 1, 6, and 12.

Measure	Group	Week 1	Week 6	Week 12
RPE	CON	2 ± 2	3 ± 1	2 ± 2
	PLA	2 ± 1	3 ± 2	2 ± 2
	MOD	3 ± 1	2 ± 2	2 ± 2
	HIGH	2 ± 2	2 ± 1	3 ± 2
Muscle soreness	CON	2 ± 1	2 ± 1	2 ± 1
	PLA	1 ± 1	2 ± 2	1 ± 1
	MOD	1 ± 1	2 ± 2	1 ± 1
	HIGH	2 ± 1	2 ± 2	1 ± 2

CON, Control; PLA, Placebo; MOD, Moderate; HIGH, High; RPE, Rating of perceived exertion; DOMS, Delayed onset muscle soreness.

### 3.8 BRUMS questionnaire

Supplementation improved vigour at week 6 (*F*[3,39] = 3.787, *p* = 0.018, ƞ_p_^2^ = 0.226; Table 8). Vigour was greater and trending towards significance in MOD (*p* = 0.081) and HIGH (*p* = 0.076) compared to CON. There was no effect of supplementation at week 6 on anger (*F*[3,39] = 0.095, *p* = 0.962, ƞ_p_^2^ = 0.007), depression (*F*[3,39] = 1.540, *p* = 0.219, ƞ_p_^2^ = 0.106), fatigue (*F*[3,39] = 1.339, *p* = 0.276, ƞ_p_^2^ = 0.093), confusion (*F*[3,39] = 0.599, *p* = 0.619, ƞ_p_^2^ = 0.044), or global mood score (*F*[3,39] = 0.612, *p* = 0.611, ƞ_p_^2^ = 0.045; Table 8).

**Table 8:** BRUMS scores for each group at weeks 1, 6 and 12.

Measure	Group	Week 1	Week 6	Week 12
Global	CON	5 ± 10	7 ± 8	7 ± 13
	PLA	6 ± 11	7 ± 9	4 ± 7
	MOD	8 ± 11	5 ± 15	7 ± 11
	HIGH	5 ± 10	3 ± 9	5 ± 13
Tension	CON	3 ± 3	3 ± 2	4 ± 3^d^
	PLA	2 ± 3	2 ± 2	2 ± 2
	MOD	4 ± 3	2 ± 3	3 ± 3
	HIGH	3 ± 3	2 ± 3	2 ± 3^a^
Anger	CON	1 ± 1	2 ± 2	2 ± 3
	PLA	1 ± 2	2 ± 1	1 ± 2
	MOD	2 ± 2	2 ± 2	1 ± 2
	HIGH	2 ± 2	2 ± 3	2 ± 4
Depression	CON	1 ± 2	1 ± 2	1 ± 2
	PLA	1 ± 2	2 ± 2	1 ± 2
	MOD	1 ± 2	2 ± 3	1 ± 2
	HIGH	1 ± 2	1 ± 2	1 ± 2
Vigour	CON	9 ± 2	6 ± 3	6 ± 4
	PLA	8 ± 3	6 ± 2	7 ± 3
	MOD	8 ± 3	9 ± 3	8 ± 3
	HIGH	8 ± 2	8 ± 2	8 ± 3
Fatigue	CON	6 ± 3	7 ± 4	8 ± 4
	PLA	7 ± 3	5 ± 3	5 ± 3
	MOD	8 ± 4	6 ± 3	5 ± 4
	HIGH	6 ± 4	5 ± 3	5 ± 4
Confusion	CON	2 ± 2	2 ± 2	2 ± 2
	PLA	2 ± 3	1 ± 2	1 ± 1
	MOD	2 ± 3	2 ± 3	1 ± 2
	HIGH	2 ± 2	1 ± 2	1 ± 3

CON, Control; PLA, Placebo; MOD, Moderate; HIGH, High; ^a^Different vs. CON; ^b^Different vs. PLA; ^c^Different vs. MOD; ^d^Different vs. HIGH. Data presented as mean ± SD.

Supplementation improved tension at week 12 (*F*[3,39] = 3.284, *p* = 0.031, ƞ_p_^2^ = 0.202; Table 8). Tension was greater in CON compared to HIGH (4 ± 3 vs. 2 ± 3, *p* = 0.022). There was no effect of supplementation at week 12 on vigour (*F*[3,39] = 0.922, *p* = 0.439, ƞ_p_^2^ = 0.066), anger (*F*[3,39] = 0.958, *p* = 0.422, ƞ_p_^2^ = 0.069), depression (*F*[1,39] = 0.260, *p* = 0.854, ƞ_p_^2^ = 0.020), fatigue (*F*[3,39] = 1.518, *p* = 0.225, ƞ_p_^2^ = 0.105), confusion (*F*[3,39] = 0.877, *p* = 461, ƞ_p_^2^ = 0.063) or global mood score (*F*[3,39] = 2.520, *p* = 0.072, ƞ_p_^2^ = 0.162; Table 8).

### 3.9 Weekly fatigue questionnaires

There was no effect of supplementation at week 6 on general fatigue (*F*[3,13] = 1.349, *p* = 0.301, ƞ_p_^2^ = 0.237), physical fatigue (*F*[3,13] = 2.155, *p* = 0.142, ƞ_p_^2^ = 0.332), emotional fatigue (*F*[3,13] = 2.809, *p* = 0.081, ƞ_p_^2^ = 0.393), mental fatigue (*F*[3,13] = 1.230, *p* = 0.338, ƞ_p_^2^ = 0.221), vigour (*F*[3,13] = 2.052, *p* = 0.156, ƞ_p_^2^ = 0.321), or total fatigue (*F*[3,13] = 0.794, *p* = 0.519, ƞ_p_^2^ = 0.155; Table 9).

**Table 9:** Weekly fatigue scores for each group at weeks 1, 6, and 12.

Measure	Group	Week 1	Week 6	Week 12
General	CON	6 ± 3	8 ± 4	7 ± 4
	PLA	7 ± 5	5 ± 5	6 ± 5
	MOD	7 ± 4	15 ± 4	13 ± 4
	HIGH	7 ± 5	6 ± 5	7 ± 4
Physical	CON	4 ± 3	7 ± 5	7 ± 4
	PLA	4 ± 2	4 ± 3	8 ± 5
	MOD	5 ± 4	15 ± 1	14 ± 5
	HIGH	4 ± 4	4 ± 5	5 ± 4
Emotional	CON	3 ± 3	4 ± 4	4 ± 5
	PLA	3 ± 3	4 ± 2	3 ± 2
	MOD	4 ± 3	11 ± 1	8 ± 1
	HIGH	3 ± 3	2 ± 3	3 ± 3
Mental	CON	5 ± 4	7 ± 5	5 ± 5
	PLA	5 ± 4	4 ± 3	3 ± 3
	MOD	6 ± 5	11 ± 3	8 ± 3
	HIGH	5 ± 4	5 ± 5	5 ± 4
Vigour	CON	11 ± 4	11 ± 3	12 ± 4
	PLA	10 ± 4	7 ± 4	8 ± 7
	MOD	11 ± 5	13 ± 5	11 ± 5
	HIGH	11 ± 4	9 ± 5	11 ± 5
Total	CON	7 ± 13	14 ± 18	12 ± 14
	PLA	9 ± 13	10 ± 11	12 ± 13
	MOD	11 ± 17	39 ± 16	33 ± 14
	HIGH	8 ± 16	8 ± 16	10 ± 12

Data presented as mean ± SD. CON, Control; PLA, Placebo; MOD, Moderate; and HIGH, High.

There was no effect of supplementation at week 12 on general fatigue (*F*[3,13] = 0.406, *p* = 0.751, ƞ_p_^2^ = 0.086), physical fatigue (*F*[3,13] = 0.673, *p* = 0.584, ƞ_p_^2^ = 0.134), emotional fatigue (*F*[3,13] = 0.861, *p* = 0.486, ƞ_p_^2^ = 0.166), mental fatigue (*F*[3,13] = 0.343, *p* = 0.795, ƞ_p_^2^ = 0.073), vigour (*F*[3,13] = 0.684, *p* = 0.578, ƞ_p_^2^ = 0.136) or total fatigue (*F*[3,13] = 0.369, *p* = 0.776, ƞ_p_^2^ = 0.079; Table 9).

## 4 Discussion

The aim of this study was to establish the impact of a pre-sleep isocaloric moderate (20 g) or high (60 g) daily dose of protein on performance, body composition and recovery indices in British Army recruits undertaking BT. Despite a *circa* 50% increase in daily protein intake and 25% increase in daily energy intake, neither isocaloric moderate or high dose protein supplementation improved markers of physical performance, body composition or chronic recovery during British Army BT. This is the first study in this population demonstrating that chronic protein supplementation does not improve performance outcomes, body composition changes and recovery. Given the lack of change in strength and FFM in all groups, it is likely the training stimulus was inadequate, limiting the impact of protein supplementation.

Protein supplementation has been consistently shown to be an effective strategy for increasing total daily protein intake and enhancing training adaptations in physically active populations, including elite sportspeople, military personnel and recreationally active individuals (9, 11, 40, 41). Ingesting 20–40 g of protein has been proposed to maximise the muscle protein synthesis (MPS) response to exercise (42) and, when consumed prior to sleep, supports MPS throughout the night (43). Protein supplementation at this time of day may be practically advantageous for military recruits due to negligible protein intakes in the evening period (13). Mechanistically, the essential amino acids, particularly L-leucine, activate the mechanistic target of rapamycin complex 1 (mTOR) which leads to translation initiation of MPS (44). Over-time, consistent elevations of MPS in response to exercise training and protein feeding supports whole-body protein balance and muscle growth (45). In the present study, both MOD and HIGH had greater total daily protein intakes following the intervention at weeks 6 and 12. Nitrogen balance, an indicator of whole-body protein balance (26), was also greater in these groups compared to CON and PLA groups, although only the HIGH group reached statistical significance. It should also be acknowledged that MPS and muscle growth is an energy demanding process. Overall, total energy intake was similar between all supplementation groups, but significantly greater compared to CON.

Despite the greater protein intake and subsequently greater positive nitrogen balance in the MOD and HIGH groups, all groups had similar improvements in performance measures. This finding contrasts with data from the United States Army which supported the use of protein supplementation over 8-weeks for improving muscular strength during training compared to a CHO placebo during BT (18, 46) which significantly improved press-up performance. Similarly, Walker et al. (46) also found protein supplementation improved press-up performance following United States Army BT. The discrepancy in findings between those studies and the current study is unknown. Historically, strength adaptations may be deemed suboptimal after 14 weeks of British Army BT (47, 48) with recruits typically making only small improvements in muscular strength (men = 7 ± 11% and women = 0 ± 10%) (48). More recently, mid-thigh pull force did not change (−0.7 ± 20.6%, *p* = 0.144) in 132 British Army recruits after 14 weeks of BT (47). Internationally, suboptimal strength adaptations have been reported during military training. For example, modest improvements in muscular strength were found in men (2.2 ± 5.9 kg) and women (3.0 ± 3.1 kg) after 8-week of Australian basic military training (49). These data indicate that there is an insufficient stimulus during BT to elicit strength adaptations (9). In addition, the cross-interference effect from concurrent training could inhibit strength adaptations (50). As protein supplementation has been shown to attenuate the interference effect (51), it is likely that the training stimulus was not sufficient to significantly increase strength. The lack of statistical differences observed between groups may also be explained in part by the large inter-individual variability in changes in strength which appears to be typical of British Army training (47). As such, the feasibility of a personalised approach to training and nutrition during military training should be explored.

All groups experienced similar changes in FFM and fat-mass with no impact of protein observed. In contrast, United States Army recruits found that 8-week of 40 g protein supplementation increased FFM (1.2 vs. 0.1 kg) compared to an isocaloric CHO placebo (15). Walker et al. (46) also reported a greater total daily protein intake and increase in FFM with protein compared to a CHO placebo (0.7 ± 1.2 vs. 0.0 ± 0.9 kg). McAdam et al. (15) reported total daily protein intakes of 2.2 ± 0.6 and 2.5 ± 0.56 g⸱kg^−1^⸱day^−1^ in the placebo and protein conditions, respectively. Despite these intakes matching and exceeding the upper limit of the current recommendations (1.5–2.0 g⸱kg^−1^⸱day^−1^) for protein intake during military training (3), a benefit on FFM was still observed (15). Based on this data, it could be that the total protein intakes in our study were not high enough to influence FFM adaptations. However, this seems unlikely given the impact protein supplementation had on nitrogen balance, indicating there was not a lack of protein availability for FFM accretion.

British Army BT has been shown to improve BMD (52). A lack of effect of protein intake on bone adaptations in this study is supported by data from the United States, with protein having no impact on markers of bone turnover during 12 weeks on U.S. Army BT (20). Notably, in this study, only the PLA group experienced no reductions in BMD and BMC in the ribs and was the only group to gain BMD and BMC at the trunk. Lower BMD and/or BMC can increase the risk of stress fracture although other factors such as age, smoking status, alcohol consumption and fitness level need to be considered (53). Supplementary energy intake has been shown to increase bone formation during military training by potentially affecting osteoblast function (54). In addition to energy, CHO feeding pre-and-post running has been shown to attenuate markers of bone resorption (55, 56), which could in-turn have chronic implications for bone turnover. Mechanistically, it is postulated that CHO feeding influences interleukin-6 activity and may regulate bone turnover via osteoclastogenesis and bone resorption (55). Although the overall changes in BMD and BMC were similar between groups in this study and did not reach statistical significance, an attenuation of BMD and/or BMC loss may have implications for stress fracture risk pertinent to this population.

There was no impact of protein supplementation on markers of chronic recovery (salivary cortisol and testosterone, mood, weekly fatigue, daily muscle soreness and daily rating of perceived exertion). Testosterone supports MPS and satellite cell activity to promote muscle recovery and growth. Conversely, cortisol has catabolic effects on skeletal muscle and its increase relative to testosterone has been associated with impaired recovery and performance (15). A mixed-macronutrient supplement attenuated declines in circulating testosterone as well as concomitant increases in cortisol in soldiers undertaking 8-week of arduous military training (16). In the United States, circulating cortisol and testosterone concentrations were shown to be similar in the initial (T:C range = 20–25) and final (T:C range = 30–35) weeks of training, with no observed benefit of protein supplementation (15). However, due to no control group being included in that study, the effects of training alone were unknown. In this study, there was a trend for higher testosterone concentrations in the latter weeks of BT in the supplemented groups compared to CON. This may indicate a beneficial impact of additional energy intake on hormonal responses during BT, although ultimately this did not improve muscular strength and FFM adaptations.

Changes in mood, perceived fatigue and perceived muscle soreness have all been used to assess the influence of nutritional intake on recovery during military training (37, 57, 58). In the current study, the only statistically significant difference observed between groups was greater tension in the final weeks of BT in CON compared to HIGH. Mechanistically, it is unclear how protein supplementation may reduce tension. Limited evidence suggests tyrosine, a non-essential amino acid and precursor to dopamine, improves cognition and mental performance in doses of 100 − 200 mg⸱kg^−1^ (59). However, although tyrosine ingestion can increase plasma concentrations, evidence of its ability to increase dopamine production is lacking (60). As such, it is possible that the reduced tension with protein supplementation in this study may have been the result of a type two statistical error (61). Overall, there was little impact of protein supplementation on mood and perceived fatigue during BT. Internationally, data in Australian recruits have shown BT programmes to support changes in mood across training weeks whereby mood improved as training progressed (58). In the United States, similar findings have been reported with greater mood disturbance in the initial weeks before improving in the following training weeks (37). Similarly, the observed lack of change over time and between groups could indicate that BT supports mood and perceived recovery of recruits. Daily RPE and DOMS were measured to assess perceived internal training load and exercise induced muscle damage. O’Leary et al. (1) demonstrated good agreement between daily RPE and heart rate-based daily training impulse (*R*^2^ = 0.57–0.77). The similar DOMS and daily RPE data between groups suggests protein supplementation did not influence either of these markers at any timepoint.

## 5 Strength and limitations

This study had several strengths that should be acknowledged. Firstly, an adequate sample size was achieved with men and women included. In addition, this study used isocaloric supplementation conditions to isolate the effects of additional protein with participants randomised to a condition. Finally, adherence to supplementation should also be acknowledged as a strength due to the research team supervising each supplementation condition. The self-report food diary method which was used in this study can underestimate total energy intake (62). It is likely that participants in this study underreported, or underestimated energy intake based on the greater estimated daily energy expenditures compared to energy intakes, and no observed reductions in total body mass. Although reductions in fat-mass were observed. It should also be acknowledged that dietary intake was only measured during specific weeks of BT. The limitations of nitrogen balance have also been acknowledged elsewhere (4). For example, nitrogen balance can underestimate the amount of protein needed to maintain a positive urinary nitrogen balance by underestimating nitrogen losses (63–65). Furthermore, the nitrogen balance method does not estimate whole-body protein synthesis compared to more sophisticated methods such as the amino acid oxidation method or the use of an oral tracer (i.e., N-glycine) (65). Nevertheless, for the purpose of this study, nitrogen balance methodology was able to demonstrate chronic changes in whole-body protein balance (26). It should also be acknowledged that this study is the result of survival bias as data were only available on those who completed BT, which will influence the magnitude of effect for any pre-post BT comparisons. Finally, protein supplementation was administered to participants in the evening prior to sleep, which has been shown to increase total daily protein intake, support MPS through the night and training outcomes (12, 41, 43). However, consuming protein during the acute post-exercise period may have more favourable effects on muscle anabolism and outcomes (66). As this study focused on the chronic effects of protein supplementation over 12 weeks of BT, future research may want to consider the impact of protein feeding on physiological adaptations around acute military training activities. The variability in the data between participants, particularly changes in strength, should be acknowledged, although this has been reported in similar British Army BT recruits (47). Finally, most participants in this study were men and it cannot be ruled out that a greater impact of protein supplementation may be observed in women.

## 6 Future work

Given that it is likely that the training stimulus was not sufficient to induce muscle growth, limiting the impact of protein supplementation, future work should explore training strategies to optimise strength adaptations in new recruits given that strength is a key determinant of occupational performance. If there are future changes to the BT programme, specifically incorporating a greater volume of resistance training, protein supplementation as a strategy to promote strength should be re-explored. Additionally, the high inter-participant variability in strength and FFM changes and nitrogen balance should also be acknowledged with future work exploring an individualised approach to protein supplementation, particularly in those with sub-optimal (1.6 g⸱kg^−1^⸱day^−1^) habitual protein intakes should be considered. Finally, the influence of nutritional (i.e., energy, CHO, calcium) supplementation on bone health during BT should also be explored given the sub-optimal changes in BMD and BMC in some skeletal regions.

## 7 Conclusion

Protein supplementation prior to sleep increased total daily protein intake and urinary nitrogen balance in British Army recruits. However, protein supplementation did not improve performance outcomes, changes in body composition and measures of chronic recovery during BT. Therefore, in the context of British Army BT, protein intakes beyond current recommendations for physically active populations to support performance, body composition and recovery does not appear to be warranted.

## Data availability statement

The raw data supporting the conclusions of this article will be made available by the authors, without undue reservation.

## Ethics statement

The studies involving humans were approved by Ministry of Defence Research Ethics Committee. The studies were conducted in accordance with the local legislation and institutional requirements. The participants provided their written informed consent to participate in this study.

## Author contributions

SC: Conceptualization, Data curation, Formal Analysis, Investigation, Methodology, Software, Writing – original draft, Writing – review & editing. JR: Conceptualization, Investigation, Writing – review & editing, Formal Analysis, Funding acquisition, Supervision. AR: Conceptualization, Investigation, Writing – review & editing, Methodology. HO: Writing – review & editing, Investigation, Methodology. RI: Writing – review & editing, Conceptualization, Funding acquisition, Supervision. LSm: Conceptualization, Funding acquisition, Supervision, Writing – review & editing. HC: Formal analysis, Methodology, Writing – review & editing. LSt: Writing – review & editing, Investigation. AR: Investigation, Writing – review & editing, Conceptualization, Funding acquisition, Supervision.
